# Supplementary material for: COZOID: contact zone identifier for visual analysis of protein-protein interactions
Source: BMC Bioinformatics. 2018 Apr 6;19:125. doi: 10.1186/s12859-018-2113-6 (PMC5889581; doi:10.1186/s12859-018-2113-6)
Supplement: Supplementary file 2 — Software build. Executable binary file of the software tool. (ZIP 118,784 kb) [file 12859_2018_2113_MOESM2_ESM.zip › cozoid/java/docs/beansbinding-1.2.1-doc.zip!/org/jdesktop/el/package-use.html]

Uses of Package org.jdesktop.el (Beans Binding)


---


|  |  |  |
| --- | --- | --- |
| **Overview** \| **Package** \| Class \| **Use** \| **Tree** \| **Deprecated** \| **Index** \| **Help** | |  |
| PREV   NEXT | **FRAMES**    **NO FRAMES**     **All Classes** |  |


---


## **Uses of Package org.jdesktop.el**

| Packages that use org.jdesktop.el | |
| --- | --- |
| **org.jdesktop.el** | Customized version of EL for Beans Binding; not for general use. |
| **org.jdesktop.el.impl** | Customized version of EL for Beans Binding; not for general use. |
| **org.jdesktop.el.impl.lang** | Customized version of EL for Beans Binding; not for general use. |
| **org.jdesktop.el.impl.parser** | Customized version of EL for Beans Binding; not for general use. |
| **org.jdesktop.el.impl.util** | Customized version of EL for Beans Binding; not for general use. |

Classes in org.jdesktop.el used by org.jdesktop.el

****BeanELResolver.BeanProperty****

****ELContext****
  
          Context information for expression evaluation.

****ELContextEvent****
  
          An event which indicates that an `ELContext` has been created.

****ELException****
  
          Represents any of the exception conditions that can arise during
expression evaluation.

****ELResolver****
  
          Enables customization of variable and property resolution behavior for EL
expression evaluation.

****Expression****
  
          Base class for the expression subclasses `ValueExpression` and
`MethodExpression`, implementing characterstics common to both.

****Expression.ResolvedProperty****

****Expression.Result****

****Expression.Result.Type****

****FunctionMapper****
  
          The interface to a map between EL function names and methods.

****MethodExpression****
  
          An `Expression` that refers to a method on an object.

****MethodInfo****
  
          Holds information about a method that a `MethodExpression`
evaluated to.

****PropertyNotFoundException****
  
          Thrown when a property could not be found while evaluating a
`ValueExpression` or `MethodExpression`.

****ValueExpression****
  
          An `Expression` that can get or set a value.

****VariableMapper****
  
          The interface to a map between EL variables and the EL expressions
they are associated with.

Classes in org.jdesktop.el used by org.jdesktop.el.impl

****ELContext****
  
          Context information for expression evaluation.

****ELException****
  
          Represents any of the exception conditions that can arise during
expression evaluation.

****Expression****
  
          Base class for the expression subclasses `ValueExpression` and
`MethodExpression`, implementing characterstics common to both.

****Expression.Result****

****ExpressionFactory****
  
          Parses a `String` into a `ValueExpression` or
`MethodExpression` instance for later evaluation.

****FunctionMapper****
  
          The interface to a map between EL function names and methods.

****MethodExpression****
  
          An `Expression` that refers to a method on an object.

****MethodInfo****
  
          Holds information about a method that a `MethodExpression`
evaluated to.

****MethodNotFoundException****
  
          Thrown when a method could not be found while evaluating a
`MethodExpression`.

****PropertyNotFoundException****
  
          Thrown when a property could not be found while evaluating a
`ValueExpression` or `MethodExpression`.

****PropertyNotWritableException****
  
          Thrown when a property could not be written to while setting the
value on a `ValueExpression`.

****ValueExpression****
  
          An `Expression` that can get or set a value.

****VariableMapper****
  
          The interface to a map between EL variables and the EL expressions
they are associated with.

Classes in org.jdesktop.el used by org.jdesktop.el.impl.lang

****ELContext****
  
          Context information for expression evaluation.

****ELException****
  
          Represents any of the exception conditions that can arise during
expression evaluation.

****ELResolver****
  
          Enables customization of variable and property resolution behavior for EL
expression evaluation.

****Expression****
  
          Base class for the expression subclasses `ValueExpression` and
`MethodExpression`, implementing characterstics common to both.

****Expression.ResolvedProperty****

****FunctionMapper****
  
          The interface to a map between EL function names and methods.

****MethodExpression****
  
          An `Expression` that refers to a method on an object.

****ValueExpression****
  
          An `Expression` that can get or set a value.

****VariableMapper****
  
          The interface to a map between EL variables and the EL expressions
they are associated with.

Classes in org.jdesktop.el used by org.jdesktop.el.impl.parser

****ELException****
  
          Represents any of the exception conditions that can arise during
expression evaluation.

****MethodInfo****
  
          Holds information about a method that a `MethodExpression`
evaluated to.

Classes in org.jdesktop.el used by org.jdesktop.el.impl.util

****ELException****
  
          Represents any of the exception conditions that can arise during
expression evaluation.

****MethodNotFoundException****
  
          Thrown when a method could not be found while evaluating a
`MethodExpression`.

****PropertyNotFoundException****
  
          Thrown when a property could not be found while evaluating a
`ValueExpression` or `MethodExpression`.

---


|  |  |  |
| --- | --- | --- |
| **Overview** \| **Package** \| Class \| **Use** \| **Tree** \| **Deprecated** \| **Index** \| **Help** | |  |
| PREV   NEXT | **FRAMES**    **NO FRAMES**     **All Classes** |  |


---
